# Baby-schema-related cues shape moral expectations about decision-makers: evidence from names, faces, and AI avatars

**DOI:** 10.3389/fpsyg.2026.1958973

**Published:** 2026-10-26

**Authors:** Zhicong Zhang, Xiuge Liu

**Affiliations:** 1 School of Educational Science, Quanzhou Normal University, Quanzhou, China; 2 School of Foreign Languages, Quanzhou Normal University, Quanzhou, China

**Keywords:** artificial intelligence, baby schema, moral dilemmas, moral predictions, social cognition

## Abstract

People routinely form expectations about how others will behave in morally difficult situations, and such expectations guide consequential decisions, including whom to trust, hire, or delegate responsibility to. The present research examined whether baby-schema-related cues systematically bias these moral predictions. Across three experiments with Chinese university students (total *N* = 858), we tested three manifestations of baby-schema-related cues that share the function of evoking infant-like associations: repeated two-syllable names (Experiment 1), babyfaced appearance (Experiment 2), and visually cute artificial intelligence (AI) avatar designs (Experiment 3). Participants consistently perceived decision-makers displaying baby-schema-related cues as less likely to make utilitarian choices involving direct harm to others. The results show that superficially irrelevant social cues can bias moral expectations about others, although the underlying psychological mechanism remains to be clarified. These findings are relevant to moral psychology, person perception, and the design and evaluation of AI systems.

## 1 Introduction

In everyday life, people are rarely confronted with moral decisions that require deliberately harming one person to save several others. Yet sacrificial moral dilemmas, most famously the trolley dilemma (Foot, 1967; Thomson, 1985), are widely used in moral psychology to study how people resolve conflicts between competing moral principles. Utilitarian reasoning weighs consequences and favors actions that maximize collective welfare, whereas deontological reasoning emphasizes duties, rights, and prohibitions against intentionally harming innocent people (Greene et al., 2004; Lucas and Galinsky, 2015). The resolution of this tension is influenced by contextual and psychological factors, including physical proximity, personal involvement, intention, and emotional responses (Alfeo et al., 2026; Bago et al., 2022; Tasso et al., 2017).

Beyond the question of how people make their own moral decisions, a separate but equally consequential question concerns how people expect others to decide. Observers constantly form predictions about the moral choices of others: whether a colleague would report misconduct, whether a doctor would prioritize one patient over another, or whether an autonomous vehicle would sacrifice its passenger to save pedestrians. Such predictions are not idle: they guide whom people trust, cooperate with, hire, or delegate responsibility to, and they increasingly shape public attitudes toward artificial intelligence (AI) systems entrusted with morally significant decisions (Awad et al., 2018). Existing evidence indicates that evaluations of moral decision-makers are shaped by the target’s social characteristics, including gender, race, socioeconomic status, and cultural background (Ellemers et al., 2019), suggesting that moral predictions are closely tied to broader processes of social perception. Yet relatively little is known about how seemingly superficial cues, such as a decision-maker’s appearance or name, shape observers’ expectations about that person’s moral choices.

### 1.1 Social perception and moral expectations

Social perception research suggests that people organize their impressions of others along two fundamental dimensions: warmth and competence (Fiske et al., 2002). Warmth captures perceived social intentions, including friendliness, trustworthiness, and harmlessness, whereas competence captures perceived ability, such as intelligence, skill, and effectiveness. These facets are directly relevant to moral judgment: the morality facet of warmth concerns whether a person is expected to act ethically, whereas the assertiveness facet of competence concerns whether a person is expected to act decisively and effectively.

Warmth and competence perceptions are also closely intertwined with moral dilemma judgments. Research on third-party judgments of moral decision-makers shows that people infer different traits from different choices: decision-makers who reject causing harm are perceived as warmer but less competent than those who accept such harm, and these inferences are shaped by the perceived affect and cognition underlying the decision (Rom et al., 2017). Whether warmth- and competence-related perceptions likewise predict observers’ expectations about others’ future moral choices remains an open question, which the present research addresses by testing whether baby-schema-related cues systematically bias moral predictions, and whether the perception measures we collected can account for that bias. Warmth and competence also map loosely onto the affective and deliberative routes emphasized in dual-process accounts of sacrificial dilemmas (Greene et al., 2004).

Which cues, then, reliably shape these social perceptions? Warmth- and competence-related impressions are formed quickly and automatically from minimal information, including facial appearance, voice, and even names (Bargh et al., 2012; Cogsdill and Banaji, 2015; Mileva, 2026; Todorov et al., 2008; Zwebner et al., 2017). Such impressions also guide consequential behavior, such as whose advice people follow (Van der Biest et al., 2025). Cues that reliably alter these perceptions may therefore also influence moral expectations. Baby schema is a natural candidate. In the present research, we use “baby schema” to refer to the infant-like feature configuration originally described by Lorenz, not to a cognitive structure held by the observer. We use “baby-schema-related cues” to refer to the specific visual or linguistic cues, such as repeated two-syllable names, babyfaced appearance, and cute AI avatar designs, that may evoke infant-like or childlike impressions. We do not assume that these cues are interchangeable indicators of a single latent schema. Rather, they serve as convergent operationalizations of cues previously associated with infant-likeness or cuteness.

Social perceivers readily draw trait inferences from minimal cues, including facial appearance and names. Facial appearance can rapidly shape impressions of warmth, competence, and trustworthiness (Todorov et al., 2008), and names can convey social expectations even in the absence of behavioral information (Zwebner et al., 2017). At the same time, these cues are often only weakly diagnostic, and in many cases normatively irrelevant, to the traits they appear to signal. A babyfaced adult may be perceived as warmer or less threatening, but this perception need not correspond to the target’s actual traits, and a repeated-syllable name reveals little about its bearer’s character. When observers nonetheless rely on such cues to infer moral character, they may form expectations that the cues themselves do not justify. These inferences are also likely to be culturally patterned. The repeated two-syllable names used in Experiment 1 are meaningful in Chinese naming practice and may not carry the same childlike or intimate connotations in other languages, and the weighting of facial cues can vary across cultural groups (Esposito et al., 2014; Lieber et al., 2025). The generality of the present findings to other languages and cultural contexts therefore remains an open question.

### 1.2 Baby schema: concept and manifestations

The concept of Kindchenschema, or baby schema, was first proposed by Lorenz (1943) to describe a cluster of infantile physical characteristics (a large head, round face, large eyes, and chubby cheeks) that tend to evoke caregiving responses. Baby schema is regarded as an innate releasing mechanism that triggers stereotypical perceptual and behavioral reactions toward targets displaying such features (Lobmaier et al., 2010; Lorenz, 1943), and exposure to these cues typically elicits perceived cuteness, an affective response associated with warmth, tenderness, and protective motivation (Glocker et al., 2009; Kawaguchi and Waller, 2024; O’Neil et al., 2026). Baby-schema-related cues influence attention, emotional responses, cognition, and behavior (Kuroda et al., 2026; Zhang and Zhou, 2020), and although cuteness has long served as an informal descriptor, it is increasingly treated as a measurable psychological construct. In the present research, we define baby schema as a set of infant-like perceptual features, spanning visual and linguistic modalities, that evoke infant-like associations and elicit perceived cuteness. We treat perceived cuteness as the proximal affective response elicited by these features not as a property of the stimuli themselves. This distinction matters because it allows the same conceptual mechanism, the activation of infant-related associations, to be tested across cues that differ greatly in surface form, from facial proportions to names to designed avatars.

Baby schema manifests across multiple modalities. The most extensively studied manifestation is the babyface: adults whose features resemble those of infants (a relatively large forehead, large eyes, a short nose, and a small chin) are perceived as more infant-like (Hwang et al., 2024; Lorenz, 1943; Zebrowitz et al., 1993), and even subtle variations such as head shape influence perceived cuteness (Alley, 1981). Neurocognitive evidence suggests that babyfaced adults are processed using mechanisms similar to those engaged by infant faces (Zebrowitz et al., 2009). Beyond facial appearance, baby schema can also be conveyed linguistically. During early language development, caregivers and infants frequently use reduplicated forms such as “night-night” and “choo-choo” (Ota et al., 2018), and the same reduplication pattern appears in repeated two-syllable names (e.g., Huahua Zhang, Bingbing Wang), which have been shown to evoke baby-schema-related perceptions (Wei et al., 2018). Finally, baby schema features can be deliberately embedded in the design of non-human agents: AI avatars and interfaces with infant-like features are perceived as cuter and elicit more positive interpersonal responses (Lieber et al., 2025; Lv et al., 2022), and similar effects are observed for brand logos and other objects containing baby-schema features (Septianto and Paramita, 2021; Tang et al., 2025; Wang et al., 2026). In the present research, we treat these three manifestations (repeated two-syllable names, babyfaced appearance, and cute AI avatar designs) as distinct cues that share the function of evoking infant-like associations. They are not interchangeable measures of a single latent construct. Rather, they provide convergent operationalizations of baby-schema-related cues, allowing us to examine whether a similar pattern emerges across modalities.

Evidence from each modality indicates that baby-schema-related cues reliably increase warmth-related perceptions, sometimes at the cost of perceived competence. Babyfaced adults are perceived as friendlier, more trustworthy, and less threatening (all components of the warmth dimension), but also as less competent and less physically capable (Berry and McArthur, 1985; McArthur and Apatow, 1984). These impressions carry real consequences: babyfaced individuals are judged less harshly in legal contexts (Berry and Zebrowitz-McArthur, 1988), are viewed as less suitable for leadership positions (Zebrowitz et al., 1991) and as less competent in political settings (Poutvaara et al., 2009), and receive more empathy and assistance in need (Lishner et al., 2008). In applied settings, babyfaced physicians generate higher patient satisfaction but are also more likely to be perceived as deceptive in malpractice cases (Chang and Chen, 2015), and warmth and competence judgments mediated the effect of babyfaced appearance on customer forgiveness in service failures (Liu and Li, 2022). Linguistic baby-schema-related cues operate similarly: repeated two-syllable names evoke perceptions of cuteness and lead observers to infer greater warmth but lower competence than non-repeated names (Wei et al., 2018), and names may even shape facial appearance over time through self-fulfilling processes (Zwebner et al., 2024). Cute design features in non-human agents likewise increase perceived warmth: visually cute AI avatars elicit greater trust, emotional attachment, and forgiveness after service failures (Cheng et al., 2020; Lee et al., 2019; Lv et al., 2021; Yim et al., 2023).

### 1.3 From baby-schema-related cues to moral expectations: possible pathways

Baby-schema-related cues may shape moral expectations through several closely related impressions, including perceived infancy, innocence, harmlessness, youthfulness, and reduced agency. The present research tests whether such cues bias moral expectations, but it does not isolate which of these impressions drives the effect. Baby-schema-related cues elicit perceived cuteness and activate broader associations with children and moral purity. Infants are prototypically “harmless” moral beings, and cues associated with infancy expand the moral circle, evoking caregiving motives and moral elevation (Kringelbach et al., 2016; Sherman and Haidt, 2011; Zhang and Zhou, 2020). Child-related cues can also activate the concept of goodness and moral purity (Desai, 2011), providing one possible reason why observers might expect that baby-schema targets will avoid causing intentional harm. These considerations lead to the prediction: decision-makers displaying baby-schema-related cues should be perceived as less likely to choose utilitarian actions that involve directly harming another person. We note, however, that because utilitarian action in sacrificial dilemmas requires active intervention, such expectations may reflect perceived passivity or low assertiveness not genuine deontological commitment.

Previous research on cuteness and morality has focused mainly on observers’ own moral behavior, for example, charitable donations, recycling, green consumption, and prosocial engagement (Huddy and Gunnthorsdottir, 2000; Jang, 2022; Lu et al., 2021; Shin and Mattila, 2021; Su et al., 2025; Wang et al., 2017; Yang et al., 2022), and on heightened moral concern for animals (Zickfeld et al., 2018), or on judgments of others’ moral transgressions (Zhang and Ye, 2023). Cuteness can even have paradoxical effects, such as when cute language style in chatbots increases consumers’ unethical behavior by enhancing their moral self-concept (Chen et al., 2025). The present research addresses a different question: whether baby-schema-related cues shape expectations about the moral choices a target decision-maker would make. This question matters because predictions about others’ choices directly inform real-world decisions about trust and delegation, and little research has examined whether baby-schema-related cues bias third-party moral predictions in sacrificial dilemmas.

### 1.4 Extension to non-human agents (AI)

Humans increasingly interact with AI systems that either make morally significant decisions or assist humans in doing so, such as autonomous vehicles and clinical decision support tools (Awad et al., 2018; Zhang et al., 2022). Many of these systems employ anthropomorphic visual designs, including cute avatars, to encourage acceptance and trust, and research on human–vehicle interaction indicates that the moral values attributed to such systems further shape users’ trust in them (Chen et al., 2023). If baby-schema-related cues shape moral expectations about human targets, the same cues may also shape expectations about AI avatars, whose visual appearance is entirely under designers’ control. Consistent with this possibility, users respond to cute AI avatars with greater warmth, empathy, and forgiveness (Cheng et al., 2020; Lv et al., 2021), suggesting that social-perceptual processes triggered by human-like features extend to designed agents.

At the same time, the extension from human to non-human agents is not automatic. People are often reluctant to attribute full mental and moral capacities (agency and the capacity for intentional, morally relevant action) to machines (Gray et al., 2007), and they are averse to delegating moral decisions to AI (Bigman and Gray, 2018). If an AI avatar is not perceived as a genuine moral decision-maker, observers’ responses may reflect judgments about its predictability or reliability not its moral tendencies. We therefore treat the extension to AI avatars as an exploratory research question not a confirmatory replication: we examine whether the pattern observed for human targets also emerges for AI avatars, while remaining agnostic about whether the underlying mechanism is identical.

### 1.5 The present study

The present research examined whether baby-schema-related cues (repeated two-syllable names, babyfaced appearance, and AI avatar designs) shape third-party predictions about decision-makers’ moral choices in a sacrificial dilemma. In all three experiments, participants read about a decision-maker who could either switch a trolley onto a side track, sacrificing one person to save five, or refrain from acting, and they indicated the perceived likelihood that the decision-maker would make the utilitarian choice. The decision-maker was identified by a repeated or non-repeated two-syllable name (Experiment 1), a babyfaced or mature face (Experiment 2), or a cute or less cute AI avatar (Experiment 3). We tested the following hypotheses:

*H1*: Individuals with repeated two-syllable names will be perceived as less likely to make utilitarian moral choices than individuals with non-repeated names.

*H2*: Individuals with babyfaced appearances will be perceived as less likely to make utilitarian moral choices than individuals with mature-faced appearances.

*H3*: AI avatars with visually cute, baby-schema-like appearances will be perceived as less likely to make utilitarian moral choices than AI avatars with less cute appearances. Because people are often reluctant to attribute full moral agency to AI systems (Bigman and Gray, 2018; Gray et al., 2007), Experiment 3 tests this prediction as an exploratory extension not a confirmatory replication.

The three experiments thus tested the same prediction across three different manifestations of baby-schema-related cues within a common experimental design. Experiment 1 established the basic effect with a purely linguistic cue; Experiment 2 replicated it with facial cues and examined whether perception measures could account for it; and Experiment 3 explored whether a similar cue-based pattern emerges for non-human AI avatars.

## 2 Experiment 1

### 2.1 Participants and design

Experiment 1 was conducted using a single factor, two-level between-participant design. Sample size was determined before any data analysis. The target sample size was set prospectively rather than determined by a formal *a priori* power analysis. Because no directly comparable effect-size estimate was available, we set a recruitment target of approximately 200 participants, which we considered a reasonable recruitment target given the exploratory nature of the effect. A total of 215 Chinese university students were recruited to participate. We excluded 10 participants, six because their completion times exceeded two standard deviations from the mean and four because they failed the attention check. Our final sample size of 205 (59.02% women, 40.98% men, *M*age = 21.71, *SD*age = 3.07, age range = 16–35) provided 80% power (α = 0.05, two-tailed) to detect an effect of *d* = 0.39, according to a sensitivity analysis (Faul et al., 2007; Zhang et al., 2022). Participants were randomly assigned to the repeated two-syllable name condition (*n* = 102, 58 women) or the non-repeated two-syllable name condition (*n* = 103, 63 women). All participants received course credit or monetary compensation (2 RMB, around 0.3 USD) for their participation.

### 2.2 Procedure and measures

Participants read the standard trolley dilemma and reported their own behavioral inclination, indicating whether they would switch the track to save five people with a single casualty or stand by and do nothing. This question concerned participants’ behavioral inclination (what they would do) not a normative judgment about what they should do. This procedure followed Zhang et al. (2022), who had participants report their own choice before judging a decision-maker. This item was included to measure participants’ own behavioral inclination so that we could examine whether participants projected their own preferences onto the target decision-maker. Because participants’ own choices were related to their predictions, own choice was included as a factor in the primary analyses. They then read a similar scenario in which a control room was added to the situation (see Figure 1). Participants were told that they were merely bystanders who could do nothing, while a track worker in the control room could switch the track, sacrificing one person to save five.

**Figure 1:**
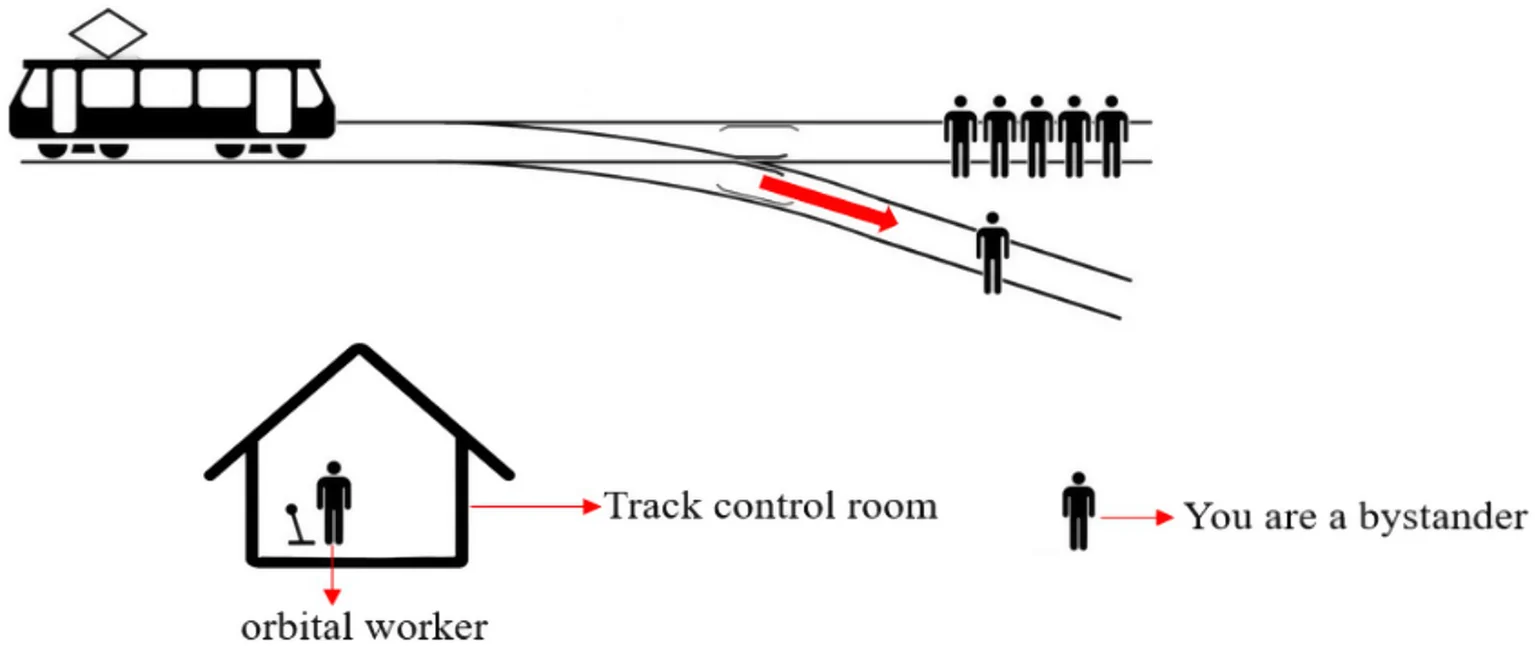
Trolley dilemma scenario in all experiments.

Participants then made judgments about one decision-maker, identified by a name. Participants were randomly assigned to see either a repeated two-syllable name (Liu Guoguo 刘果果) or a non-repeated two-syllable name (Liu Guo 刘果). The names were taken from Wei et al. (2018). Following previous research (Argo et al., 2010), participants silently recited the name to activate speech perception.

As a manipulation check, participants rated how childlike or adult-like the person they imagined from the name seemed, on a 7-point scale (1 = most baby-like, 7 = most adult-like); lower scores indicated stronger baby schema perception (Gorn et al., 2008; Wei et al., 2018). This manipulation check was intended to assess participants’ perception of the person implied by the name not the name itself, although cue-based and target-based impressions cannot be fully separated in this design. Participants also rated their familiarity with the name on a 7-point scale and then indicated the percentage likelihood (0 = very unlikely, 100 = very likely) that the track worker would switch the track, sacrificing one person to save five (the utilitarian choice).

To improve data quality, we restricted the device type and IP address in all experiments, and we included attention checks that required participants either to choose a specific response or to leave a question unanswered. Data from participants who failed an attention check were excluded. At the end of the assessment, participants completed a real-person verification to prevent automated responses.

### 2.3 Results

#### 2.3.1 Manipulation check

An independent-samples *t*-test was conducted to examine whether participants perceived decision-makers with repeated two-syllable names as more baby-like than those with non-repeated two-syllable names. The analysis revealed a significant difference between the two conditions. Participants rated repeated two-syllable names (*M* = 4.22, *SD* = 1.36) as more baby-like than non-repeated two-syllable names (*M* = 5.61, *SD* = 1.39), *t*_(203)_ = −7.27, *p* < 0.001, *d* = 1.01, 95% CI [−1.77, −1.02]. Because lower scores reflected greater resemblance to babies, these results suggest that repeated two-syllable names successfully increased perceptions of baby-likeness.

Although this manipulation checks primarily assessed categorical perceptions related to infancy (i.e., baby-like versus adult-like) not directly measuring affective reactions such as perceived cuteness, associations with infancy constitute a core component of linguistic baby schema (Wei et al., 2018). The findings therefore support the effectiveness of the linguistic baby schema manipulation based on repeated two-syllable names. In addition, no significant difference in name familiarity was found between the two conditions, *t*(203) = 0.49, *p* = 0.627, *d* = 0.068, indicating that familiarity was unlikely to confound the results.

#### 2.3.2 Analysis of expected moral choices

We examined whether the name condition influenced participants’ expectations about the decision-maker’s moral choice. Because participants’ own choices in the initial trolley dilemma were strongly related to their predictions (135 of 205 participants, 65.9%, chose the utilitarian option), we conducted a two-way analysis of variance (ANOVA) with experimental condition (repeated vs. non-repeated two-syllable name) and participants’ own moral choice (utilitarian vs. deontological) as between-participants factors. The main effect of experimental condition was significant, *F*(1, 201) = 14.35, *p* < 0.001, $\eta_{p}^{2}$ = 0.07, 95% CI [−14.86, −4.69]: participants perceived the decision-maker with a repeated two-syllable name (adjusted *M* = 59.55) as less likely to make a utilitarian choice than the decision-maker with a non-repeated two-syllable name (adjusted *M* = 69.32). The main effect of participants’ own moral choice was also significant, *F*(1, 201) = 45.56, *p* < 0.001, $\eta_{p}^{2}$ = 0.18, 95% CI [12.33, 22.50]: participants who chose the utilitarian option themselves were more likely to expect the decision-maker to make a utilitarian choice. The interaction between the two factors was not significant, *F*(1, 201) = 2.17, *p* = 0.14, $\eta_{p}^{2}$ = 0.01.

As a supplementary analysis, we compared the two conditions without controlling for participants’ own moral choices. Participants perceived the decision-maker with a repeated two-syllable name (*M* = 63.71, *SD* = 20.90) as having a lower chance of making a utilitarian choice than the decision-maker with a non-repeated two-syllable name (*M* = 70.97, *SD* = 17.49), *t*(203) = −2.70, *p* = 0.008, *d* = 0.38, 95% CI [−12.57, −1.96]. To examine the robustness of this effect, we repeated the ANOVA while controlling for gender and age. Age was included as a conservative covariate because age may be associated with moral-dilemma judgments and with sensitivity to baby-schema-related cues. The main effect of experimental condition remained significant, *F*(1, 199) = 15.82, *p* < 0.001.

### 2.4 Discussion

The findings of Experiment 1 supported Hypothesis 1. Participants perceived decision-makers with repeated two-syllable names as less likely to switch the track and sacrifice one person to save five others than decision-makers with non-repeated two-syllable names. In other words, individuals associated with repeated two-syllable names were viewed as less likely to make utilitarian choices. One possible explanation is that repeated two-syllable names were perceived as baby-schema-related cues (Wei et al., 2018), which is consistent with the observed difference in moral predictions participants made about the decision-maker. This interpretation is consistent with the manipulation check, which showed that participants perceived repeated two-syllable names as significantly more baby-like.

The present experiment did not directly assess interpersonal impressions such as warmth, trustworthiness, or gentleness, and the subsequent experiments did not support a warmth-based account of the effect. The current results also suggest that participants’ own moral preferences may influence how they infer the likely behavior of others in moral dilemmas (Zhang et al., 2022). More specifically, participants who favor deontological responses may be more likely to project these preferences onto decision-makers associated with baby-schema-related cues, expecting them to act in accordance with similar moral principles.

Experiment 1 thus provides initial evidence that linguistic baby-schema-related cues conveyed through repeated two-syllable names can influence moral predictions about decision-makers. However, it remains unclear whether similar effects would emerge for visual baby-schema-related cues, such as babyfaced appearances, and whether these effects are driven by specific interpersonal perceptions, particularly warmth. The following experiments addressed these questions.

## 3 Experiment 2

### 3.1 Participants and design

Experiment 2 was conducted using a single factor, two-level between-participant design. Experiment 2 also used a prospective sample-size target rather than a formal *a priori* power analysis. Experiment 2 used the same general recruitment principle but aimed for a larger sample because it included additional measures and exploratory mediation analyses. We recruited 333 Chinese university students. We excluded 24 participants, nine because their completion times exceeded two standard deviations from the mean and 15 because they failed the attention check. A sensitivity test (Faul et al., 2007; Zhang et al., 2022) showed that our final sample size of 307 (65.8% women, 34.2% men, *M*age = 20.75, *SD*age = 2.71) provided 80% power (α = 0.05, two-tailed) to detect an effect of *d* = 0.32. We randomly assigned participants to one of two face conditions (babyface = 154 vs. mature face = 153). All participants received monetary compensation.

### 3.2 Procedure and measures

The materials and procedure were similar to Experiment 1, except that the names were replaced with pictures of human faces taken from Gorn et al. (2008), namely the two face stimuli (babyfaced and mature faced), with only the image clarity adjusted and no other modification. The original images are shown in that article, to which readers are referred. Participants first read the standard trolley dilemma and indicated whether they would switch the track to save five people with a single casualty or stand by and do nothing. They then read the same scenario again, in which the decision-maker in the control room was identified by a photograph (babyface vs. mature face). Participants rated the target on perceived maturity, trustworthiness, expertise, perceived babyfaceness, empathy, competence (smart, efficient, wise), and warmth (kind, friendly, sincere), each on a 7-point scale (higher scores indicated greater perceived maturity, trustworthiness, expertise, babyfaceness, empathy, competence, and warmth). Trustworthiness referred to a general impression of honesty and integrity, which is conceptually closer to benevolence than to competence-based reliability. Competence was measured separately with the items smart, efficient, and wise.

These perception measures were included to examine whether the effect of baby-schema-related cues on moral predictions could be accounted for by the warmth and competence dimensions of social perception, and by exploratory attributes relevant to the stimulus domain. Participants then indicated the percentage likelihood (0 = very unlikely, 100 = very likely) that the target would switch the track, sacrificing one person to save five (the utilitarian choice).

### 3.3 Results

#### 3.3.1 Manipulation check

Results revealed that the babyfaced person (*M* = 3.92, *SD* = 1.62) was perceived as having more of a babyface than the mature-faced person (*M* = 2.48, *SD* = 1.25), *t*(305) = 8.70, *p* < 0.001, *d* = 0.99, 95% CI [0.76, 1.23]. The babyfaced person was also perceived as warmer (*M* = 4.75 vs. 4.33; *t*(305) = 2.87, *p* = 0.004, *d* = 0.33, 95% CI [0.10, 0.55]) and as more empathetic (*M* = 4.72 vs. 4.42; *t*(305) = 2.16, *p* = 0.031, *d* = 0.25, 95% CI [0.02, 0.47]) than the mature-faced person. The two faces did not differ significantly in perceived trustworthiness (*t*(305) = 1.87, *p* = 0.062, *d* = 0.21, 95% CI [−0.01, 0.44]) or perceived expertise [*t*(305) = 0.11, *p* = 0.909]. These results indicate that the babyface manipulation was successful. Across the full sample, maturity and babyfaceness ratings were negatively correlated (*r* = −0.20), and only 14.3% of participants rated the target above the scale midpoint on both dimensions. This overlap was more common in the babyface condition (25.3%) than in the mature-face condition (3.3%). The two perceptions therefore largely came apart, though some overlap remained.

#### 3.3.2 Analysis of expected moral choices

We then tested whether the face condition influenced participants’ expectations about the decision-maker’s moral choice. Because participants’ own choices in the initial trolley dilemma were related to their predictions (163 of 307 participants, 53.1%, chose the utilitarian option), we conducted a two-way ANOVA with experimental condition (babyface vs. mature face) and participants’ own moral choice (utilitarian vs. deontological) as between-participants factors. The main effect of experimental condition was significant, *F*(1, 303) = 40.59, *p* < 0.001, $\eta_{p}^{2}$ = 0.12, 95% CI [−16.85, −8.90]: participants perceived the babyfaced decision-maker (adjusted *M* = 55.05) as less likely to make a utilitarian choice than the mature-faced decision-maker (adjusted *M* = 67.92). The main effect of participants’ own moral choice was also significant, *F*(1, 303) = 39.08, *p* < 0.001, $\eta_{p}^{2}$ = 0.11: participants who chose the utilitarian option themselves were more likely to expect the decision-maker to make a utilitarian choice. The interaction between the two factors was not significant, *F*(1, 303) = 1.06, *p* = 0.30, $\eta_{p}^{2}$ = 0.003.

As a supplementary analysis, we compared the two conditions without controlling for participants’ own moral choices. Participants perceived the babyfaced decision-maker (*M* = 56.19, *SD* = 19.31) as having a lower chance of making a utilitarian choice than the mature-faced decision-maker (*M* = 67.75, *SD* = 17.97), *t*(305) = −5.43, *p* < 0.001, *d* = 0.62, 95% CI [−15.75, −7.37]. A two-way ANOVA additionally controlling for gender and age yielded the same pattern, *F*(1, 301) = 36.70, *p* < 0.001.

We additionally examined whether the perception measures could account for the effect of facial type on moral predictions. Mediation analyses (PROCESS Model 4; Hayes, 2022) with gender, age, and participants’ own moral choice as covariates showed that perceived warmth was positively (not negatively) associated with the expected likelihood of utilitarian choice (*b* = 3.77, *p* < 0.001), and perceived competence did not differ between conditions (*p* = 0.97) and did not mediate the effect (indirect effect = 0.01, 95% CI [−0.88, 0.78]). Because warmth was related to utilitarian expectations in the direction opposite to the hypothesized mechanism, warmth could not explain why babyfaced targets were expected to make fewer utilitarian choices, and no other perception measure (trust, empathy) provided a consistent explanation. The hypothesized warmth-mediated mechanism was therefore not supported.

### 3.4 Discussion

Experiment 2 replicated the main effect of Experiment 1 using facial stimuli: babyfaced decision-makers were perceived as less likely to make the utilitarian choice than mature-faced decision-makers. Consistent with prior research, babyfaced targets were also perceived as warmer (Berry and McArthur, 1985), but this perception did not account for the observed bias: perceived warmth was positively, not negatively, associated with expected utilitarian action. Thus, the effect of baby-schema-related cues on moral predictions does not appear to reflect a warmth-based mechanism. Instead, it may reflect broader expectations associated with baby-schema-related cues, such as perceived passivity or low assertiveness, an interpretation we consider in the General Discussion, although this possibility was not directly tested. The similar pattern across names and faces should not be taken as evidence that participants organized these cues into a single psychological schema. The two experiments show only that two different baby-schema-related cues produced similar patterns in moral prediction. Whether participants conceptually group repeated names with babyfaced targets could be tested more directly, for example by asking them to pair names with faces.

Because people increasingly interact with AI systems, we asked whether a similar cue-based pattern emerges for non-human agents. Many of these systems use visual designs intended to encourage user acceptance, and we therefore examined whether the pattern observed for human targets also emerges for AI avatars. To keep the comparison clear and avoid implying interactive human-AI communication, Experiment 3 used static visual representations of AI, not interactive assistants. In this study, we use the term AI avatar to refer to the static visual representation of this autonomous AI system. No human operator was mentioned or depicted. Experiment 3 thus asked whether the influence of visual baby-schema-related cues on moral predictions also applies to non-human AI avatars.

## 4 Experiment 3

### 4.1 Participants and design

Experiment 3 used a two-condition between-participant design. Its sample-size target was set prospectively rather than by a formal *a priori* power analysis. Because Experiment 3 also included additional measures and exploratory analyses, we aimed for a larger sample than in Experiment 1. We recruited 375 Chinese university students. We excluded 29 participants, 12 because their completion times exceeded two standard deviations from the mean and 17 because they failed the attention check. A sensitivity test (Faul et al., 2007; Zhang et al., 2022) showed that our final sample size of 346 (68.5% women, 31.5% men, *M*age = 20.82, *SD*age = 2.87) provided 80% power (α = 0.05, two-tailed) to detect an effect of *d* = 0.30. We randomly assigned participants to the high-cuteness condition (*n* = 177) or the low-cuteness condition (*n* = 169). All participants received monetary compensation.

### 4.2 Procedure and measures

The materials and procedure were similar to Experiment 2, except that the human faces were replaced with pictures of AI avatars taken from Lv et al. (2022). The original images are shown in that article. Participants first completed the same moral dilemma task, indicating whether they would switch the track to save five people with a single casualty or stand by and do nothing. They then read the same scenario again, in which the decision-maker in the control room was represented by an AI avatar. Participants rated the avatar on cuteness, seriousness, coldness, and expertise, each on a 7-point scale, and also rated it on perceived warmth (kind, friendly, sincere) and competence (smart, efficient, wise); higher scores indicated greater perceived cuteness, seriousness, coldness, expertise, warmth, and competence.

As in Experiment 2, these perception measures were included to examine whether the effect could be accounted for by social-perceptual impressions. Seriousness and coldness were included because cute AI avatars may simultaneously appear less serious or less emotionally cold, impressions that could plausibly affect expectations about action in a high-stakes dilemma. Finally, they indicated the percentage likelihood (0 = very unlikely, 100 = very likely) that the AI avatar would switch the track, sacrificing one person to save five (the utilitarian choice).

### 4.3 Results

#### 4.3.1 Manipulation check

Manipulation checks confirmed that the high-cuteness AI avatar was perceived as cuter (*M* = 5.14 vs. 3.86; *t*(344) = 7.69, *p* < 0.001, *d* = 0.83, 95% CI [0.61, 1.05]) and warmer (*M* = 4.56 vs. 3.70; *t*(344) = 5.43, *p* < 0.001, *d* = 0.58, 95% CI [0.37, 0.80]) than the low-cuteness avatar. The low-cuteness avatar was perceived as more serious (*M* = 3.96 vs. 3.29; *t*(344) = −3.85, *p* < 0.001, *d* = 0.41, 95% CI [−0.63, −0.20]) and colder (*M* = 4.16 vs. 3.36; *t*(344) = −4.36, *p* < 0.001, *d* = 0.47, 95% CI [−0.68, −0.26]) than the high-cuteness avatar. The two avatars did not differ significantly in perceived expertise [*t*(344) = 0.34, *p* = 0.733] or perceived competence [*t*(344) = 1.28, *p* = 0.202]. These results indicate that the avatar cuteness manipulation was successful and produced impressions consistent with baby-schema-related design.

#### 4.3.2 Analysis of expected moral choices

We tested whether avatar cuteness influenced participants’ expectations about the AI decision-maker’s moral choice. Because participants’ own choices in the initial trolley dilemma were related to their predictions (207 of 346 participants, 59.8%, chose the utilitarian option), we conducted a two-way ANOVA with experimental condition (high vs. low cuteness) and participants’ own moral choice (utilitarian vs. deontological) as between-participants factors. The main effect of experimental condition was significant, *F*(1, 342) = 12.36, *p* < 0.001, $\eta_{p}^{2}$ = 0.035, 95% CI [−14.02, −3.96]: participants perceived the high-cuteness AI avatar (adjusted *M* = 62.81) as less likely to make a utilitarian choice than the low-cuteness avatar (adjusted *M* = 71.80). The main effect of participants’ own moral choice was also significant, *F*(1, 342) = 6.44, *p* = 0.01, $\eta_{p}^{2}$ = 0.02: participants who chose the utilitarian option themselves were more likely to expect the AI decision-maker to make a utilitarian choice. The interaction between the two factors was not significant, *F*(1, 342) = 1.97, *p* = 0.16, $\eta_{p}^{2}$ = 0.006.

As a supplementary analysis, we compared the two conditions without controlling for participants’ own moral choices. Participants perceived the high-cuteness AI avatar (*M* = 63.21, *SD* = 24.37) as having a lower chance of making a utilitarian choice than the low-cuteness avatar (*M* = 72.37, *SD* = 22.46), *t*(344) = −3.63, *p* < 0.001, *d* = 0.39, 95% CI [−14.12, −4.20]. Because Levene’s test indicated unequal variances across the condition cells (*W* = 4.60, *p* = 0.004), we also conducted a Welch’s *t*-test, which yielded a consistent result, *t*(343.6) = −3.64, *p* < 0.001. A two-way ANOVA additionally controlling for gender and age yielded the same pattern, *F*(1, 340) = 13.21, *p* < 0.001.

As in Experiment 2, we examined whether the perception measures could account for the effect of avatar cuteness on moral predictions. Perceived warmth was positively, not negatively, associated with the expected likelihood of utilitarian choice (*b* = 2.32, *p* < 0.001), and perceived competence did not differ between conditions (*p* = 0.20); the warmth and competence indirect effects were 1.87 (95% CI [0.39, 3.80]) and 0.58 (95% CI [−0.31, 1.88]), respectively, so neither could explain the effect in the hypothesized direction. An exploratory examination of the remaining perception measures found no robust mediator. Perceived seriousness showed a pattern directionally consistent with the effect (the high-cuteness avatar was perceived as less serious, and seriousness was positively associated with expected utilitarian action), but the indirect effect was marginal (indirect effect = −1.00, 95% CI [−2.55, 0.00]) and should be interpreted with caution. The hypothesized warmth-mediated mechanism was therefore not supported.

### 4.4 Discussion

Experiment 3 explored whether a similar cue-based pattern emerges for non-human agents. Consistent with Experiments 1 and 2, participants perceived AI avatars with cute, baby-schema-like appearances as less likely to make utilitarian choices than avatars with less cute appearances. As in Experiment 2, perceived warmth was elevated by the baby-schema manipulation but did not explain the effect, and no perception measure provided a robust mediation account. These results suggest that a similar cue-based pattern can emerge for designed, non-human agents, although the underlying mechanism remains to be clarified. However, because the instructions described the target as an AI system and we did not verify whether participants perceived it as autonomous, the effect may reflect impressions of a human target, not overgeneralization to an autonomous AI system.

## 5 General discussion

The present research examined how baby-schema-related cues conveyed through names, facial features, and AI avatar designs influence people’s moral expectations about decision-makers in dilemma situations. Across three experiments, a consistent pattern emerged: decision-makers associated with baby-schema-related cues were perceived as less likely to make utilitarian choices involving the sacrifice of one person to save five others. This pattern appeared for human targets in Experiments 1 and 2 and for AI avatars in Experiment 3 (see Figure 2). Although baby-schema-related cues also elevated perceived warmth in Experiments 2 and 3, warmth was positively, not negatively, associated with expected utilitarian action, and no perception measure accounted for the observed bias. Finally, the rates of first-person utilitarian responding in the Chinese sample (65.9% in Experiment 1, 53.1% in Experiment 2, and 59.8% in Experiment 3) were lower than some previous reports for switch-type dilemmas. This may partly reflect the behavioral framing of our item, which asked what participants would do, not what they should do or what would be morally permissible. Cultural variation and differences in sample composition may also have contributed (Bago et al., 2022).

**Figure 2:**
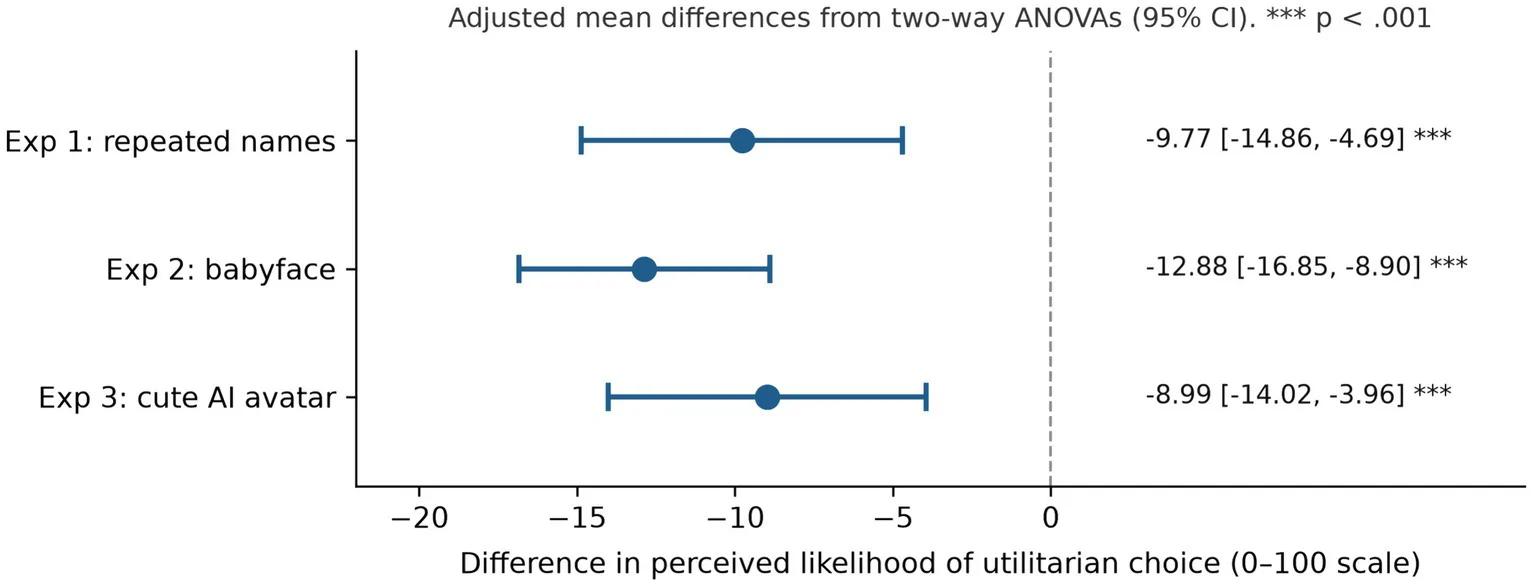
Adjusted mean differences in the perceived likelihood of utilitarian choice across Experiments 1–3, with 95% confidence intervals. Negative values indicate that decision-makers displaying baby-schema-related cues were expected to be less likely to make the utilitarian choice. ***p < 0.001.

These findings contribute to a broader understanding of how superficial social cues shape moral inference. The study focused not on individuals’ actual moral decisions but on observers’ expectations about those decisions. Participants were not evaluating real acts of moral reasoning; instead, they inferred likely behavior on the basis of socially meaningful cues associated with baby schema. The findings therefore reflect a social-cognitive process grounded in impression formation and heuristic judgment.

These findings also extend work showing that both adults and children draw trait inferences from minimal perceptual information such as faces and names. Whereas much of that work has focused on impressions of warmth, competence, trustworthiness, or leadership, the present research shows that similar cues can also shape predictions about how a target will act in a morally consequential situation. This suggests that superficial appearance and identity cues can reach beyond person evaluation and into expectations about moral behavior.

The present findings help constrain possible mechanisms underlying these expectations. Contrary to the warmth-based account we initially considered, the observed bias was not explained by perceived warmth: baby-schema-related cues increased perceived warmth, yet warmth was positively (not negatively) related to expected utilitarian action, and perceived competence did not differ between conditions. The observed bias may be related to associations with infancy, innocence, or harmlessness, although these possibilities were not directly assessed in the present study (Kringelbach et al., 2016; Sherman and Haidt, 2011). Observers may expect baby-schema-related targets to avoid causing intentional harm. At the same time, because utilitarian action in the present dilemma requires active intervention, such expectations may partly reflect assumptions about passivity or low assertiveness not genuine deontological commitment. An exploratory analysis in Experiment 3 was consistent with this possibility. Cute AI avatars were perceived as less serious, and perceived seriousness was positively associated with expected utilitarian action. However, the indirect effect was marginal and should be interpreted cautiously. Distinguishing deontological expectations from expectations of inaction remains an important task for future research (Gawronski et al., 2017).

The present results can also be situated within the dual-process framework described above. Baby-schema-related cues increased perceived warmth, but warmth did not account for the shift toward deontological expectations. These findings therefore do not provide direct support for a warmth-mediated dual-process account. They do, however, underscore the value of separating affective impressions, such as warmth, from more deliberative impressions, such as competence, when studying moral expectations.

The present findings also point to a broader bias in social perception. Participants relied on highly superficial cues, such as repeated names or visually cute avatars, to infer moral tendencies and ethical values. This pattern is consistent with the babyface overgeneralization effect (Zebrowitz, 1997), whereby adaptive responses originally associated with infant care are generalized to unrelated adult or artificial targets. Although such heuristics may facilitate rapid social judgments, they can also produce systematic biases by encouraging people to infer moral character from cues that are largely unrelated to actual ethical behavior.

### 5.1 Implications of the present research

The current findings extend previous work on the relationship between baby schema and morality. Earlier studies suggested that baby-schema-related cues broaden the moral circle by increasing caregiving tendencies and moral elevation among observers (Lu et al., 2021; Sherman and Haidt, 2011). The present study further demonstrates that these cues also shape expectations regarding the target’s likely moral choice in a sacrificial dilemma. Specifically, individuals associated with baby-schema-related cues were expected to be less likely to engage in harm-based utilitarian action, although perceived harmlessness was not directly measured.

The findings also have practical implications for the design of AI avatars and related ethical guidelines. AI systems are increasingly used in domains such as autonomous driving, healthcare, and customer service, where users may form moral expectations about how these systems should behave (Zhang et al., 2022). The current results suggest that such expectations are influenced by visual appearance as much as by system functionality. Designers may therefore intentionally or unintentionally shape users’ moral perceptions through avatar design. Such effects are not limited to perceptions: cute avatars in a university whistleblowing system, for instance, reduced students’ fear of retaliation and increased reporting intentions. In light of prior work, AI agents with stronger baby-schema-related cues may be perceived as more empathetic and trustworthy in contexts emphasizing care and emotional support, whereas more neutral or mature appearances may better fit contexts requiring impartial or utilitarian decision-making. At the same time, these findings raise ethical concerns regarding the deliberate use of appearance-based cues to influence user trust and moral perception, particularly when these cues are unrelated to the AI system’s actual decision-making processes. These issues may inform future ethical frameworks for AI development (Paraman and Anamalah, 2023). These implications assume that participants treated the target as an autonomous AI system. Because we did not directly verify this interpretation, the findings should be applied cautiously to trust in autonomous AI systems, not to human-operated interfaces.

Methodologically, the findings point to the importance of controlling target characteristics in moral psychology research. The observed effects of facial appearance, names, and avatar design on moral predictions suggest that seemingly peripheral stimulus features may introduce systematic variance into experimental paradigms. Researchers examining moral prediction should therefore consider target appearance and identity as potential confounding variables and adopt appropriate counterbalancing procedures where possible.

### 5.2 Limitations and future direction

Although the present study provides initial evidence that baby-schema-related cues influence moral expectations, several limitations should be considered when interpreting the findings. These limitations also point to several important directions for future research.

One important limitation is that the current experimental design cannot clearly distinguish between expectations of deontological moral orientation and expectations of behavioral inaction. Baby-schema-related cues are commonly linked to lower perceived agency, submissiveness, and reduced assertiveness, as well as to warmth. As a result, targets displaying baby-schema-related cues may be perceived as hesitant, passive, or unlikely to intervene in high-conflict situations. Because utilitarian action in the trolley dilemma requires active intervention, participants’ expectations that baby-schema-related targets would avoid switching the track may partly reflect assumptions about passivity not genuine deontological commitment. Consistent with this interpretation, perceived warmth did not account for the observed bias in the present research, and perceived seriousness (a proxy for decisiveness) showed a directionally consistent but marginal pattern in Experiment 3. This issue limits the interpretability of the present findings. Future studies could address this problem by applying process dissociation (PD) procedures (Conway and Gawronski, 2013) or the CNI model (Consequences, Norms, Inaction; Gawronski et al., 2017), which allow different motivational tendencies to be separated analytically. In particular, the CNI model may help clarify whether baby-schema-related cues increase expectations of norm adherence specifically or simply increase expectations of inaction regardless of moral consequences. Future research could also incorporate additional mediating variables, such as perceived agency, dominance, or decisiveness, alongside warmth and competence.

A second limitation concerns the specificity of the experimental manipulations. In Experiment 1, repeated two-syllable names were used as linguistic baby-schema-related cues. However, these naming patterns may also convey other social meanings, including childishness, intimacy, femininity, or lower authority, particularly within certain cultural contexts. Similarly, Experiment 2 relied on a relatively small number of facial stimuli. Without larger stimulus sets and appropriate random-effects analyses, it is difficult to fully exclude the possibility that the observed effects were partly influenced by idiosyncratic properties of the selected faces, such as attractiveness, empathy, or perceived credibility, not baby schema alone. Future research should therefore employ larger and more systematically validated stimulus sets, including computer-generated faces that manipulate baby schema features while controlling for other facial characteristics and semantic associations. Such approaches would improve both internal validity and generalizability.

Another limitation is that the present study relied exclusively on a single impersonal trolley dilemma. Because only one moral scenario was used, it remains unclear whether the observed effects generalize across different types of moral conflict. Future research should examine a wider range of moral contexts, including highly personal dilemmas such as the footbridge dilemma, as well as more ecologically valid situations involving medical ethics, organizational decision-making, or everyday interpersonal conflicts (Greene et al., 2008). In addition, individual and cultural differences in responses to baby-schema-related cues deserve further attention. Previous studies suggest that sensitivity to baby schema may vary according to factors such as gender and implicit attitudes toward infants (Ikeda, 2026), hormonal state (Lobmaier et al., 2015; Sprengelmeyer et al., 2009), and cultural background (Esposito et al., 2014; Lieber-Milo, 2025). Research has also shown that excessively strong baby schema features in AI or robotic agents may sometimes reduce perceived trustworthiness, depending on the interaction context (Chen and Jia, 2023), and that cuteness perception can also arise from interaction contexts beyond static baby schema features (Ohashi and Nittono, 2026). These findings suggest that the influence of baby schema on moral expectations is likely to be context-dependent, not universally positive. Future studies could therefore examine how different forms of AI agents, such as humanoid systems or voice-only assistants, interact with users’ expectations to shape moral judgments.

A further limitation concerns perceived age. Baby-schema-related cues are closely associated with youth, and participants may have inferred that targets with repeated two-syllable names or babyfaced appearances were younger than their counterparts. Because perceived age was not measured, we cannot rule out the possibility that age-related expectations, not baby-schema perception per se, contributed to the observed effects. The manipulation check was intended to capture baby-likeness, not chronological age, and its wording has been clarified accordingly. Future research should measure perceived age directly and test whether it mediates the effects.

Experiment 3 also leaves open how participants mentally represented the AI entity. Although we described the target as an AI avatar and used a static image, we did not include a manipulation check asking whether participants interpreted it as an autonomous AI decision-maker, an AI assistant, or a visual representation of a human operator. If participants inferred that a human was operating the avatar, the observed effect could partly reflect impressions of that human target. Relatedly, because people are often reluctant to attribute moral agency to machines (Gray et al., 2007; Bigman and Gray, 2018), participants may not have accepted that an AI avatar could make a moral decision in the trolley scenario. Their responses may therefore have reflected suspension of belief or compliance, not genuine expectations. Future research should directly measure perceived moral agency, autonomy, and plausibility, and compare AI targets that are clearly autonomous with those presented as human-operated.

Finally, the neural mechanisms underlying these moral prediction processes remain unclear. Previous neuroimaging research has shown that baby schema perception activates brain regions such as the amygdala and medial orbitofrontal cortex (Glocker et al., 2009), areas that are also involved in moral cognition (Greene and Haidt, 2002). Future work could investigate how neural responses to superficial social cues, including baby-schema-related visual or auditory features, interact with the neural processes involved in evaluating others’ moral tendencies. Such research could clarify the biological and cognitive mechanisms underlying these social judgments.

## 6 Conclusion

In conclusion, the present research provides convergent evidence that baby-schema-related cues (repeated two-syllable names, babyfaced appearance, and AI avatar designs) systematically shape observers’ expectations about others’ moral decisions. Across three experiments, decision-makers associated with these cues were consistently perceived as less likely to make utilitarian choices in a sacrificial dilemma. Perception measures (perceived warmth, competence, and related attributes) did not account for this bias: warmth was positively, not negatively, associated with expected utilitarian action, and no robust mediator was identified. The findings show a reliable cue-based bias in moral expectations, while leaving open whether this bias reflects associations with infancy, innocence, harmlessness, perceived youth, reduced agency, or generalized expectations of inaction.

## Data Availability

The datasets presented in this study can be found in online repositories. The names of the repository/repositories and accession number(s) can be found at: https://osf.io/q5g7f/.
