# Supplementary material for: Baby-schema-related cues shape moral expectations about decision-makers: evidence from names, faces, and AI avatars
Source: Front Psychol. 2026 Oct 26;17:1958973. doi: 10.3389/fpsyg.2026.1958973 (PMC13746296; doi:10.3389/fpsyg.2026.1958973)
Supplement: Supplementary file 1 [file Supplementary_file_1.DOCX]

**Supplementary Materials（附录材料）**

*The materials were administered in Chinese. The symbol "\" indicates the two between-subject versions (Experiment 1: repeated two-syllable name 刘果果/Liu Guoguo vs. non-repeated name 刘果/Liu Guo).*

**Instructions（指导语）**

中文（Chinese）：您好，欢迎参与本次研究。接下来您将会看到一个道德判断的场景，根据问题作出自己的回答。请根据自己看到问题后的第一感觉做出选择，不要过多考虑现实情况。选择没有正确和错误之分，请放心作答。本研究不会收集个人信息，研究结果仅做科研之用。感谢您的配合。

英文（English）：Hello and welcome to this study. Next you will be presented with a moral judgment scenario and make your own response based on the question. Please make your judgment based on your first feelings after seeing the question, and do not think too much about the reality of the situation. There is no right or wrong choice, so please feel free to answer. No personal information will be collected in this study, and the results will be used for scientific research only. Thank you for your cooperation.

**Demographics（人口学信息）**

中文（Chinese）：1. 您的性别：男 / 女；2. 您的年龄：____

英文（English）：1. What is your gender? (Man / Woman) 2. What is your age? ______

**Moral dilemma scenario（电车困境情境）**

中文（Chinese）：一个疯子把五个无辜的普通人绑在电车轨道上。一辆失控的无人驾驶的有轨电车朝他们驶来，并且片刻后就要碾压到他们。幸运的是，你可以拉一个拉杆，让电车开到另一条轨道上。但是还有一个问题，那个疯子在那另一条轨道上也绑了一个无辜的普通人。如果是你站在拉杆旁，你是否会扳动拉杆让电车撞向一个人，拯救五个人。（1 扳动拉杆 / 2 不扳动拉杆）

英文（English）：A madman has tied five innocent people to the tram tracks. An out-of-control driverless tram is heading toward them and will run them over in a few moments. Fortunately, you can pull a lever to send the tram onto another track. However, the madman has also tied one innocent person to that other track. If you were standing next to the lever, would you pull it to make the tram run over one person and save five? (1 = pull the lever, 2 = do not pull the lever)

**Experiment 1（实验1）**

**Third-person scenario（第三人称情境）**

中文（Chinese）：还是遇到上面的情况，而你只是一个旁观者目睹了这一状况。在轨道附近有一个控制室，里面有一名工作人员，名叫刘果果\刘果。他可以选择拉杆让电车开到另一条轨道上。请根据你的第一感觉回答下面问题。

英文（English）：Still facing the situation above, but you are just a bystander witnessing the situation. There is a control room near the track that houses a track worker named Liu Guoguo\Liu Guo. He has the option of pulling a lever to make the tram go to another track. Please answer the following questions based on your first sense.

**Attention check（注意力检测）**

中文（Chinese）：6. 这名工作人员叫什么 [注意力检测]（1 李宇 / 2 马伟 / 3 刘果果\刘果）

英文（English）：What is the staff member's name? [attention check] (1 Li Yu / 2 Ma Wei / 3 Liu Guoguo\Liu Guo)

**Manipulation check（操纵检验）**

中文（Chinese）：请你在脑海中想象一下刘果果\刘果这个人。在现实生活中，有些名字听起来更像孩子的名字，有些名字听起来更像成年人的名字。根据这个名字，刘果果\刘果这个人看起来更像儿童还是更像成人？（1 = 更像儿童，7 = 更像成人）

英文（English）：Please imagine the person Liu Guoguo\Liu Guo in your mind. In real life, some names sound more like children's names, and some sound more like adults' names. Based on the name, does the person Liu Guoguo\Liu Guo seem more like a child or more like an adult? (1 = more child-like, 7 = more adult-like)

**Dependent measure（因变量）**

中文（Chinese）：8. 你认为刘果果\刘果有多大可能性扳动轨道使车辆撞向一个人，拯救五个人（输入0(非常不可能)到100(非常可能)的数字）

英文（English）：How likely do you think Liu Guoguo\Liu Guo is to pull the lever so that the vehicle hits one person and saves five? (Enter a number from 0 = very unlikely to 100 = very likely)

**Experiment 2（实验2）**

**Third-person scenario（第三人称情境）**

中文（Chinese）：依旧还是遇到上面的情况，你只是一个旁观者目睹了这一状况。在轨道附近有一个控制室，里面有一名工作人员可以拉杆让电车开到另一条轨道上。这名工作人员的证件照如下图所示，以下图片都为同一个人。

英文（English）：Still facing the situation above, and you are just a bystander witnessing the situation. There is a control room near the track that houses a track worker who has the option of pulling a lever to make the tram go to another track. The identification photos of the staff member are shown below (the images are all of the same person).

**Rating items（评价条目）**

中文（Chinese）：9-12. 请评价这名工作人员的形象与特质（成熟度、信任度、娃娃脸程度、专业度、温暖度、能力、同理心；均为 7 点量表）

英文（English）：Please evaluate the staff member on perceived maturity, trustworthiness, babyfaceness, expertise, warmth, competence, and empathy (each on a 7-point scale).

**Attention check（注意力检测）**

中文（Chinese）：注意力检测：本题请勿做任何选择，直接点击提交

英文（English）：[attention check] Please do not make any choices on this question; just click the Submit button at the end of the question.

**Dependent measure（因变量）**

中文（Chinese）：13. 你认为这名工作人员有多大可能性扳动轨道使车辆撞向一个人，拯救五个人（输入0(非常不可能)到100(非常可能)的数字）

英文（English）：How likely do you think this staff member is to pull the lever so that the vehicle hits one person and saves five? (Enter a number from 0 = very unlikely to 100 = very likely)

**Experiment 3（实验3）**

**Third-person scenario（第三人称情境）**

中文（Chinese）：依旧是遇到之前的情况，你也是一个旁观者目睹了以上情况。不同的是这是由人工智能系统控制的一个无人驾驶的电车，人工智能可以控制轨道和电车转向。这个人工智能的虚拟形象及名称如下图所示，以下图片都为同一个形象。

英文（English）：You are a bystander witnessing a situation like the one above. The difference is that this is an unmanned tram controlled by an AI system, which controls the tracks and steering of the tram. The virtual image and name of this AI are shown below (the images are all of the same image).

**Rating items（评价条目）**

中文（Chinese）：14-16. 请评价这个人工智能的形象与特质（可爱度、严肃度、冷酷度、专业度、温暖度、能力；均为 7 点量表）

英文（English）：Please evaluate this AI on cuteness, seriousness, coldness, expertise, warmth, and competence (each on a 7-point scale).

**Attention check（注意力检测）**

中文（Chinese）：注意力检测：本题请选择 3，然后继续完成后续题目

英文（English）：[attention check] Please select 3 for this question and continue to complete the following questions.

**Dependent measure（因变量）**

中文（Chinese）：17. 你认为这个人工智能有多大可能性会扳动轨道使车辆撞向一个人，拯救五个人（输入0(非常不可能)到100(非常可能)的数字）

英文（English）：How likely do you think this AI is to pull the lever so that the vehicle hits one person and saves five? (Enter a number from 0 = very unlikely to 100 = very likely)
